# Supplementary material for: Catabolism of acetosyringone and co-metabolic transformation of 2,4,6-trichlorophenol by a novel FAD-dependent monooxygenase
Source: mSystems. 2026 Jan 27;11(2):e01242-25. doi: 10.1128/msystems.01242-25 (PMC12911418; doi:10.1128/msystems.01242-25)
Supplement: Supplemental Material — Supplemental methods, figures, and tables. [file msystems.01242-25-s0001.pdf]

Catabolism of Acetosyringone and Co-metabolic Transformation of 2,4,6-Trichlorophenol by a Novel FAD-dependent Monooxygenase

Tomas Engl[1], Lydie Jakubova[1], Zdena Skrob[2], Stephanie Campeggi[1], Roman Skala[1], Magdalena Folkmanova[1], Petr Pajer[3], Martin Chmel[3,4], Tomas Cajthaml[2], Michal Strejcek[1], Jachym Suman[1], Ondrej Uhlik[1]

[1]*University of Chemistry and Technology, Prague, Faculty of Food and Biochemical Technology, Department of Biochemistry and Microbiology, Prague, Czech Republic*

[2]*Institute of Microbiology, Academy of Sciences of the Czech Republic, Prague, Czech Republic*

[3]*Military Health Institute, Military Medical Agency, Prague, Czech Republic*

[4]*Department of Infectious Diseases, First Faculty of Medicine, Charles University and Military University Hospital Prague, Prague, Czech Republic*

Supplementary information

Methods

Mineral medium solution

The mineral medium (MM) was composed of solution A (109.55 g $Na_2HPO_4 \cdot 12H_2O$, 27 g KH_2PO_4, 10 g $(NH_4)_2SO_4$ in 1000 ml distilled water, pH 7–7.5, autoclave-sterilized), solution B (0.3 g $Ca(NO_3)_2$ dissolved in 50 ml distilled water, filter-sterilized), solution C (2 g $MgSO_4$ dissolved in 50 ml distilled water, filter-sterilized), and solution D (0.1 g $FeSO_4$ dissolved in 50 ml distilled water, filter-sterilized) where 900 ml of sterile distilled water was initially mixed with 100 ml of solution A and then 5 ml of solutions B, C and D was added. Minimal medium (MM) was stored at 4 °C for short-term use; otherwise, it was freshly prepared prior to each experiment.

Stock solutions

Acetosyringone (AS) was obtained from Sigma-Aldrich (Germany), as were the other substrates and pollutants unless stated otherwise. The AS stock solution for cultivation was prepared at a concentration of 0.1 M in ethanol for UV spectroscopy (Lach-Ner, Czech Republic) and filter-sterilised.

To reduce the number of samples required during the initial screening of the transformation potential of the bacterial consortium ASC12 toward selected aromatic pollutants (APs) and chlorophenols (CPs), mixtures of pollutants were prepared. All pooled pollutant solutions were prepared by mixing stock solutions of individual pollutants, each at a concentration of 2 mM in DMSO (Lach-Ner, Czech Republic), in order to minimise potential synergistic toxicity effects. The pollutants (all Sigma-Aldrich, Germany unless stated otherwise) were grouped as follows: i) phenol (Lach-Ner, Czech Republic), 2-chlorophenol (2-CP), 2,6-dichlorophenol (2,6-DCP), 2,4,6-trichlorophenol (2,4,6-TCP), ii) 3-chlorophenol (3-CP), 3,5-dichlorophenol (3,5-DCP(, 2,3,4,5-tetrachlorophenol (2,3,4,5-TeCP), iii) 4-chlorophenol (4-CP), 2,4-dichlorophenol (2,4-DCP), 2,4,5-trichlorophenol (2,4,5-TCP), pentrachlorophenol (PCP), iv) bisphenol A, diethyl ether, and v) dibutyl phthalate, diphenyl phthalate. Remaining pollutants, namely naphthalene, biphenyl, phenanthrene, and dibenzofuran, were analysed individually.

For the individual-compound setup, stock solutions of the tested pollutants, namely phenol, 2-CP, 3-CP, 4-CP, 2,6-DCP, 3,5-DCP, 2,4-DCP, 2,4,6-TCP, 2,4,5-TCP, 2,3,4,5-TeCP, and pentaCP, were individually prepared by dissolving each compound in DMSO to a final concentration of 10 mM.

Stock solutions of selected lignin building-blocks, namely AS, caffeic acid, coumaric acid, eugenol, ferulic acid (Sigma-Aldrich, Japan), guaiacol, orcinol, protocatechuate, dihydroxyphenylacetate, 3,4-dihydroxybenzoic acid, sinapic acid, vanillic acid, vanillin and veratric acid (MCE, USA) were individually prepared by dissolving each compound in DMSO to obtain final concentrations of 10 μM; 0,1 mM; 1 mM; 2 mM and 3 mM and 10 mM.

HPLC-PDA analysis

Briefly, the samples were analysed by reverse-phase HPLC (Shimadzu Nexera XR equipped with SPD-M20A DAD) using an Arion 5 μm C18 Plus 100x4.6 column with a flow rate of 0,8 ml/min at 40 °C. Mobile phase A consisted of deionised water, and phase B was HPLC-grade methanol, both containing 0.02% (v/v) trifluoracetic acid (Supelco, Germany). The retention times and working concentration range (0−500 μM) of all tested compounds were determined by analysing standards of corresponding pollutants. All the analyses were performed by the following method: 0−2 min: 8−25 % B; 2−6 min: 25−90 % B; 6−9 min: 90 % B; 9−10.5 min: 90−25 % B; 10.5−14 min: 25−8 % B. Integration of peaks and post-run analysis was conducted in Shimadzu's LabSolution software.

Quality control and assembly of the sequencing data

Quality control of Illumina short reads was performed using bbduk.sh v39.06 (parameters: ktrim=r k=23 mink=11 hdist=1 tpe tbo qtrim=r trimq=10 ftm=5; https://sourceforge.net/projects/bbmap/) with the BBMap-supplied adapters.fa file. For Nanopore long reads, adapter trimming and quality filtering were conducted using Porechop v0.2.4 (default settings; (1)) and Filtlong v0.2.1 (--min_length 1000 --keep_percent 90; https://github.com/rrwick/Filtlong).

Assembly of the sequencing data was carried out using two strategies: Flye v2.9.4 (2) for long-read-only assembly, and hybridSPAdes (3) v4.0.0 for hybrid assembly of both short and long reads. Contigs from individual assemblies were binned into metagenome-assembled genomes (MAGs) using SemiBin2 v2.1.0 (multi_easy_bin mode) (4). MAG quality was assessed using CheckM2 v1.0.2 (5), and dereplication was performed with CoverM v0.7.0 (cluster --ani 99 --quality-formula Parks2020_reduced) (6). MAGs were functionally annotated using the NCBI Prokaryotic Genome Annotation Pipeline (2024-07-18.build7555) (7) and taxonomically classified with GTDB-Tk v2.4.0 (8).

The expression of candidate genes

Cells from 50 ml of the O/N culture induced with 0.3mM IPTG were harvested by centrifugation, washed twice with MMS, and resuspended in 5ml 50 mM NaH2PO4, 300 mM NaCl, pH = 8, and immediately used for enzyme activity assay. Enzyme active assays were conducted similarly to RCAs described above, with some modifications. Briefly, suspensions of induced cells were washed and adjusted to $OD_{600} = 10$ in MM, as a biotic control, *E. coli* BL21(DE3) cells bearing an empty pET19b plasmid were used. For the enzyme activity assays, 250 µl of the cell suspension (OD_{600} = 10) was incubated with 2.5 µl of 10 mM control substrate: vanillin, ferulic acid, vanillic acid, protocatechuate, dihydroxyphenylacetate, and AS chosen based on the annotated function of the candidate genes, at 12 °C for 48 hours with shaking. Similarly, all candidate genes were also tested for their ability to deplete 2,6-DCP and 2,4,6-TCP. The reactions were stopped with methanol, processed by vortexing, sonication, and centrifugation. The resulting supernatants were transferred to HPLC vials for subsequent HPLC-PDA analysis. All samples were prepared in triplicate.

The preparation of the AS1-derived biosensor strain and the induction assay

The pTr-egfp backbone and IR DNA sequence were PCR-amplified and fused employing the NEBuilder HiFi DNA Assembly Master Mix (New England Biolabs, USA), following the manufacturer's protocol. *E. coli* DH5α transformants were selected on solid LB plates amended with 2 µg/ml of tetracycline. The sequence of the resultant plasmid pTr-IR-egfp was verified by Sanger sequencing, and it was inserted into AS1 cells by electroporation (Choi et al., 2006) (9). Transformants were selected on LB medium with tetracycline (2 µg/ml). Transformants were transferred to fresh LB plates with tetracycline, and the presence of the plasmid pTr-IR-egfp in transformed cells was verified by PCR using isolated plasmid DNA (QIAprep Spin Miniprep Kit, Qiagen). For the induction assay, the AS1 cells bearing pTr-IR-egfp (hereinafter referred to as AS1/egfp) were cultivated O/N in liquid LB medium with 2 µg/mL tetracycline. Cells were washed twice (10 minutes/5000 × rcf/18°C) and resuspended in MM 1× to reach a final OD_{600} of 0.1. The cell suspension was supplemented with 2 µg/mL tetracycline and distributed in 200 µL aliquots into a pure Grade S flat- and clear-bottom black microtiter plate. Wells were then supplemented with 2 µL of either a substrate stock solution diluted in DMSO or pure DMSO (control wells). The substrates tested were 3,4-dihydroxybenzoic acid, acetosyringone, caffeic acid, eugenol, ferulic acid, guaiacol, *p*-coumaric acid, orcinol, sinapic acid, vanillic acid, vanillin, and veratric acid in stock concentrations of 10 µM; 0,1 mM; 1 mM; 2 mM, and 3 mM. The microtiter plates were incubated in a Synergy H1 Plate Reader (BioTek) at 28°C with continuous agitation for 96 hours. OD_{600} and eGFP fluorescence at 476 nm

(excitation)/520 nm (emission) were measured at 1-hour intervals. To quantify the induction capacity of each substrate, R ratios were obtained by normalizing the eGFP fluorescence values by OD_{600} readings for each well and time point, and the induction rate expressed as the fold-induction relative to control wells (i.e., only DMSO added) was calculated. To rule out potential interference from the substrates or their transformation products, AS1/wild-type cells cultivated in plain LB medium were treated analogously alongside AS1/egfp. The induction rates of AS1/egfp were then compared to the corresponding values obtained from the AS1/wild-type cells to yield the relative induction rate (RI).

Identification of the initiator of AS transformation within the asdBCAD gene cluster

To test the transformation of AS by induced recombinant *E. coli* BL21(DE3) strains expressing individual *asd* genes, a procedure similar to the RCA assay described in the main text was employed. Briefly, 250 µl of cell suspension (OD_{600} = 10) was added to 4 ml glass vials, followed by the addition of 2.5 µl of a 10 mM AS stock solution. As a control, *E. coli* BL21(DE3) harbouring an empty expression vector was used. All reactions were prepared in triplicate. Incubations were carried out at 28 °C and 150 rpm for 24 hours. To terminate the reactions, 250 µl of HPLC-grade methanol was added to each vial. Before HPLC-PDA analysis, cells were removed by centrifugation at maximum speed for 3 minutes, and the supernatants were transferred to fresh glass vials.

The LC-MS analysis specifications

The cell suspensions were acidified by the addition of 20 µL of 1 M HCl and extracted with an aliquot of ethyl acetate (EtOAc, 2 mL; 5 times). The extracts were then dried with sodium sulphate and concentrated to dryness using a nitrogen stream. Samples were diluted in 1 mL of methanol and analysed with LC-MS. The separation of analyses was performed in a Kinetex Polar-C18 2.6 µm 100 Å chromatographic column (100 x 2.1 mm, Phenomenex, CA, USA). The mobile phase was composed of 0.1% formic acid in water and 0.1% formic acid in methanol. The flow rate was 0.4 ml/min, the column temperature was maintained at 40°C, and the mobile phase gradient elution started with 5% methanol for 3.0 min and increased up 100% in 15 min. After 5 min at 100% methanol, the starting conditions were established, and the column was equilibrated for 5 min prior to injection of the next sample. 10 µl of sample extract was injected. For the non-target analysis, both positive and negative modes were used. QTOF MS was tuned using Swarm Autotune for the mass range m/z 50–1600. For the positive mode, reference m/z = 121.050873 and 922.009798 were used during the analysis to achieve the best

mass accuracy. Subsequently, for negative mode, reference m/z = 112.985587 and m/z= 980.016375 were used. A single MS mode (ESI, both polarities, 5 spectra/s) in the range 50–1600 m/z was chosen to perform metabolite profiling on the cell extracts. For MS/MS analysis, an autoMS/MS mode was used. Spectra were acquired using two different collision energies (CE = 20 and 40 eV) and a fragmentor voltage of 110 V. The mass range was 50–1000 m/z, and the MS/MS acquisition rate was 5 spectra/s. To confirm the data, a targeted MS/MS method was used. For AS in positive mode, for 2,4,6-TC/ in negative mode; set up with collision energies (CE = 10, 30, and 40 eV) and fragmentor voltage 110 V in both polarities. The resulting fragmentation spectra and isotopic pattern were compared with the Metlin spectra library (https://metlin.scripps.edu/landing_page.php?pgcontent=mainPage).

The proportional transformation product amounts were calculated based on peak area as the relative concentration of AS or 2,4,6-TCP equivalent in the extracts.

Supplementary Figures

i)

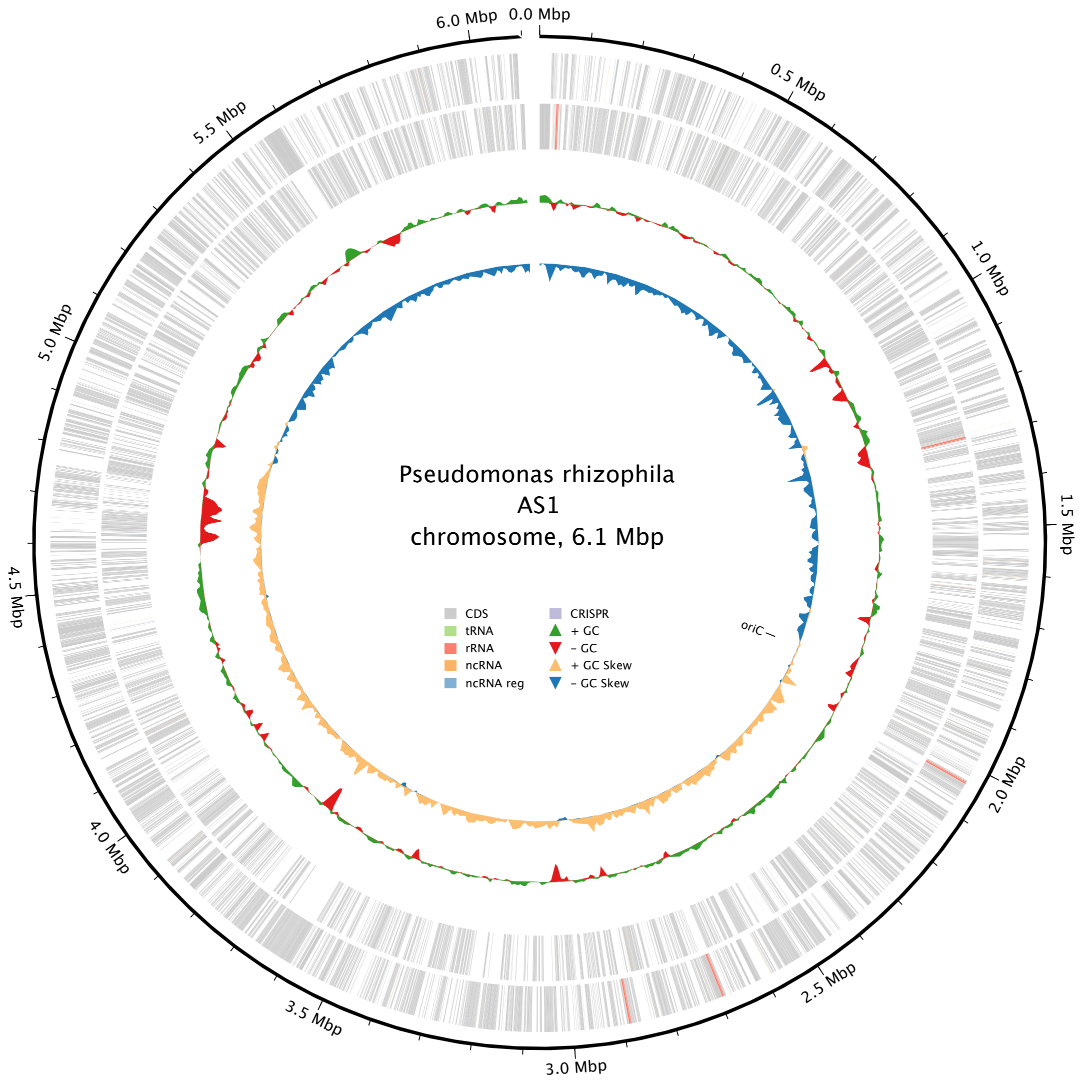

ii)

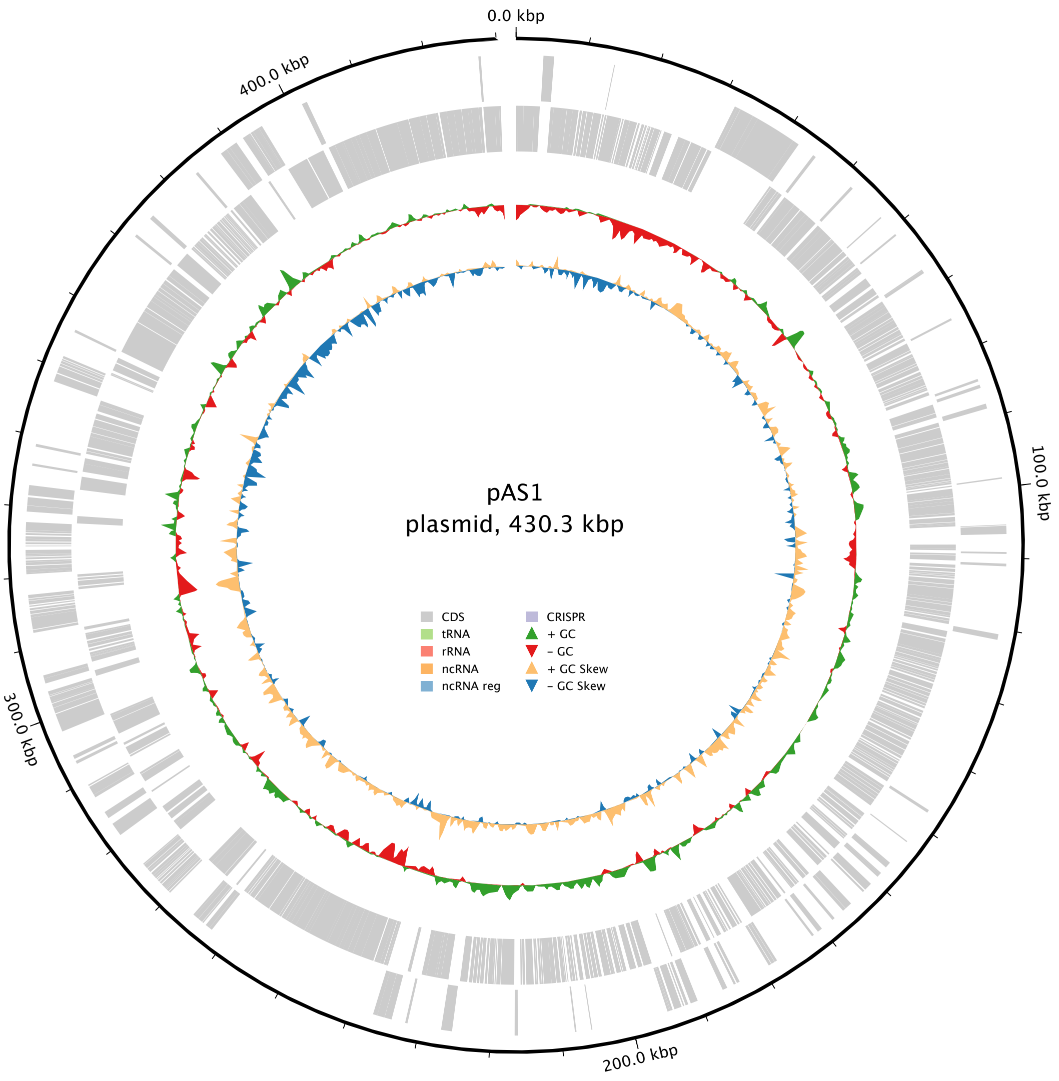
0.0 kbp
100.0 kbp
200.0 kbp
300.0 kbp
400.0 kbp
pAS1
plasmid, 430.3 kbp
CDS
tRNA
rRNA
ncRNA
ncRNA reg
CRISPR
+ GC
- GC
+ GC Skew
- GC Skew

iii)

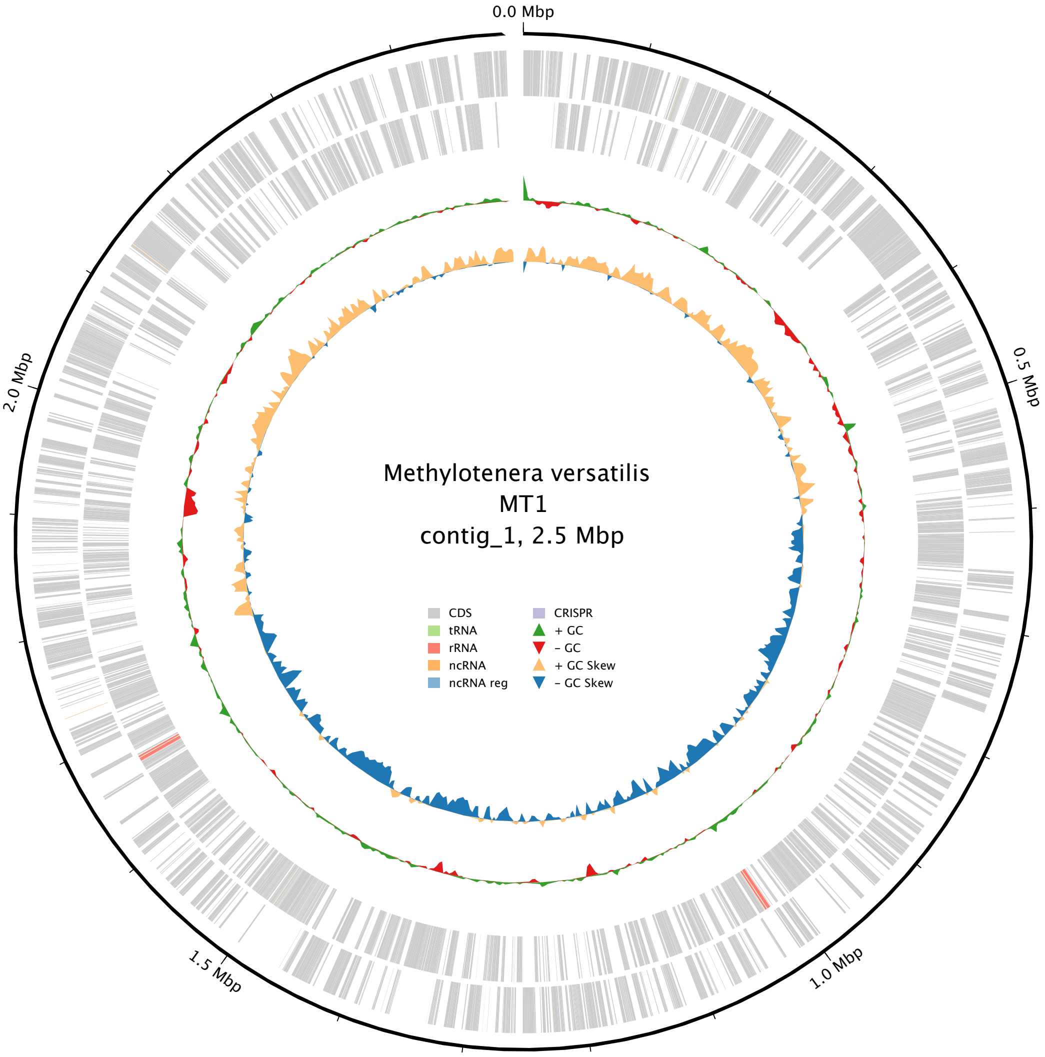

Figure 1: Graphical representation of the metagenome-assembled genomes (MAGs) obtained after sequencing the ASC12 consortium, including common genomic parameters: i) MAG of *Pseudomonas rhizophila* AS1, ii) plasmid MAG of *Pseudomonas rhizophila* AS1 (pAS1), and iii) MAG of *Methylotenera versatilis* MT1.

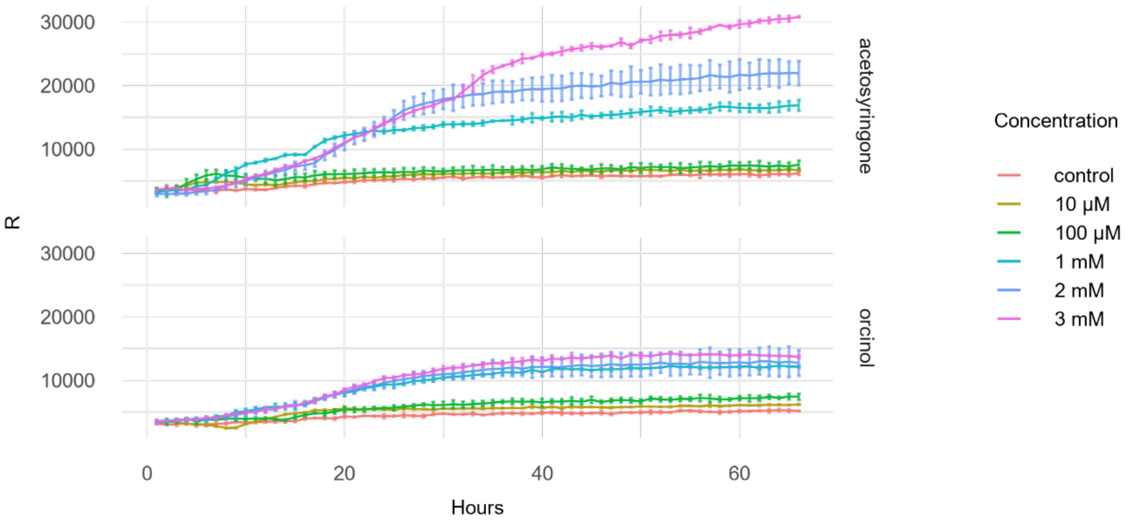

Figure 2: Exemplary results showing the induction of the intergenic region (P_*IR*) in AS1 by acetosyringone and orcinol. R represents the ratio of relative fluorescence units to optical density at 600 nm (R = RFU/OD600). R values are plotted with standard deviation as error bars (two replicates).

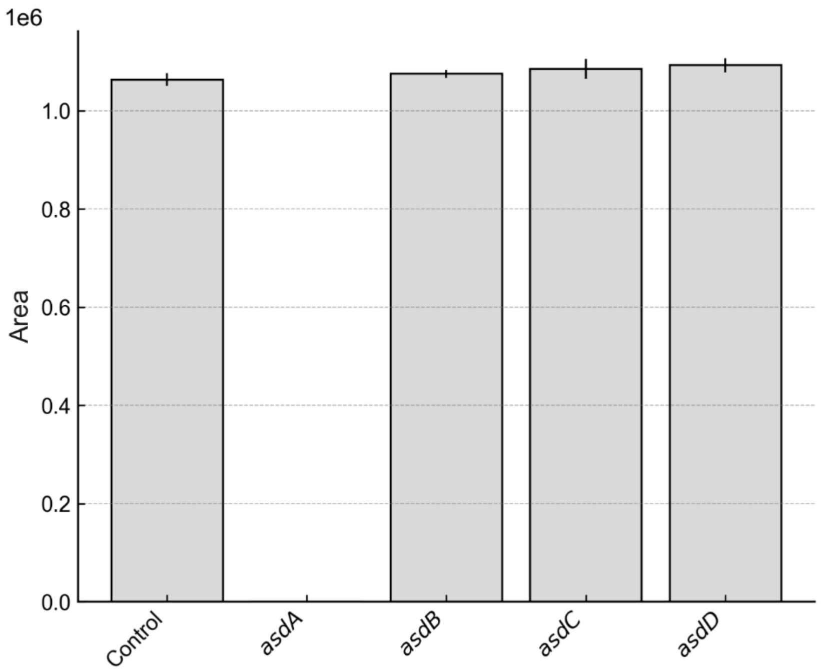

Figure 3: Acetosyringone (AS) transformation by induced recombinant *E. coli* BL21(DE3) strains expressing individual *asd* genes. The graph shows the depletion of AS by *E. coli* BL21(DE3) cells carrying plasmids with *asdA*, *asdB*, *asdC,* or *asdD*. A strain harbouring an empty vector was used as a control. The Y-axis represents the peak area, corresponding to the residual amount of acetosyringone in the sample. Error bars represent the standard deviation from biological replicates.

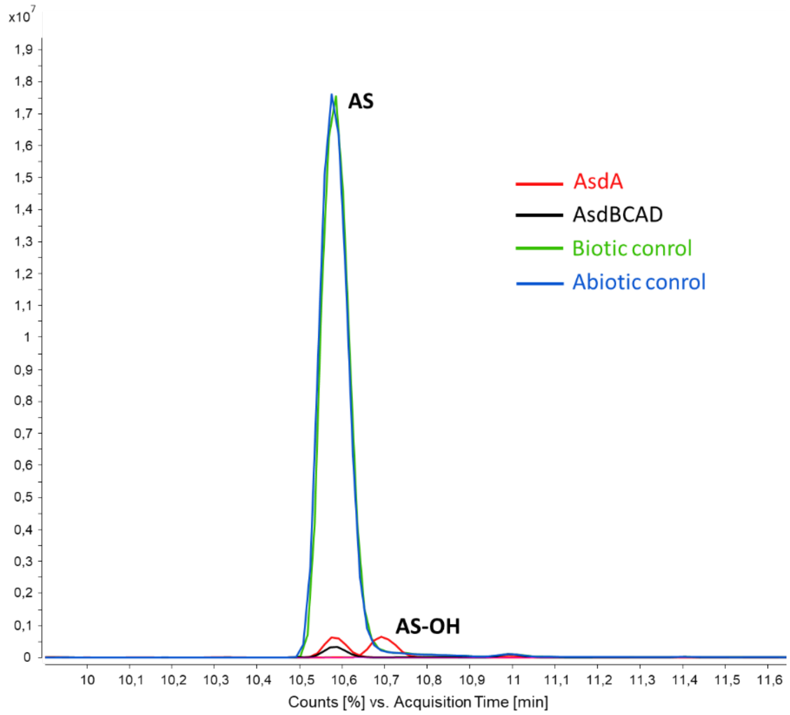

Figure 4: Chromatogram showing the transformation of AS (missing peak of AS in black and red spectra) and the detection of its transformation product (AS-OH) by *E. coli* cells expressing only the gene *asdA* (in black), which is missing in the cells expressing the whole *asdBCAD* gene cluster (red).

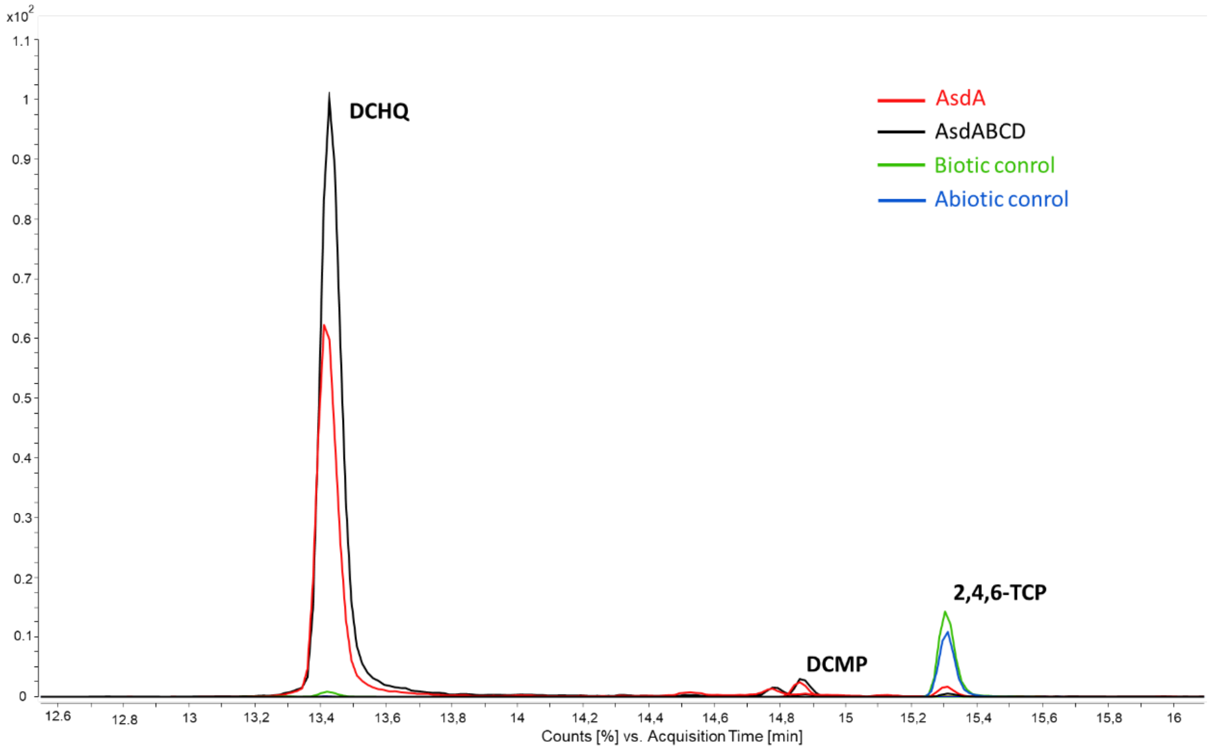

Figure 5: Chromatogram showing the transformation of 2,4,6-trichlorophenol (2,4,6-TCP) and the detection of its transformation products, namely, 2,6-dichlorohydroquinone (DCHQ) and 2,6-dichloro-4-methoxyphenol (DCMP) by *E. coli* cells expressing either only the gene *asdA* (in red) or the *asdBCAD* gene cluster (black), which are missing in the cells with the empty vector (biotic control) and abiotic controls (green and blue, respectively).

Supplementary Table

Supplementary Table 1: Primers used in this study

Product	Designation	5'-3' sequence *	Amplicon length [bp]
16S rRNA gene	8F	AGAGTTTGATCMTGGCTCAG	1501
	1509R	GYTACCTTGTTACGACTT	
	515F	GTGYCAGCMGCNGCGG	411
	926R	CCGYCAATTYMTTTRAGTTT	
pET-19b backbone	pET_F	ctagcataaccccttggggcctc	5576
	pET_R	ggtatatctccttcttaaagttaaac	
pTr backbone	pUCP_F	atggtgagcaagggcgagg	6926
	pUCP_R	acgcatcctcacgataataag	
asdABCD cluster	asdA_F	AGAAGGAGATATACCatgaacactcatgctgatg	4722
	asd_R	AAGGGGTTATGCTAGtcagaatgggaaggagaattg	
asdA coding sequence	asdA_F	AGAAGGAGATATACCatgaacactcatgctgatg	1284
	asdA_R	AAGGGGTTATGCTAGtcagaggaactgagagcgtc	
	vdh_F	AGAAGGAGATATACCatgctggacgtgcccctgttg	1479

vdh coding sequence (vanillin dehydrogenase)	vdh_R	AAGGGGTTATGCTAGtcagatcgggtaatgccgg	
fcs coding sequence (feruoyl CoA-synthetase)	fcs_F	AGAAGGAGATATACCatgagtgttgagttcagatc	1899
	fcs_R	AAGGGGTTATGCTAGctagttcgcccgcaggatcg	
ech coding sequence (hydoxycinnamoyl-CoA hydratase/lyase)	ech_F	AGAAGGAGATATACCatgagcaattacgaaggtcgc	861
	ech_R	AAGGGGTTATGCTAGtcagcgcttgtaggtctgc	
vanAB cluster (vanillate *O*-demethylase)	van_F	AGAAGGAGATATACCatgtatcccaagaacacctg	2054
	van_R	AAGGGGTTATGCTAgatatccaacaccaaaagtg	
pcaHG cluster (protocatechuate 3,4-dioxygenase)	pca_F	AGAAGGAGATATACCatgactgacaagcctggttatc	1303
	pca_R	AAGGGGTTATGCTAGtcagtagtcgaagaacaccg	
hpaD coding sequence (3,4-hydroxyphenyl acetate 2,3-dioxygenase)	hpa_F	AGAAGGAGATATACCatgggcgaagtcgtcctggc	888
	hpa_R	AAGGGGTTATGCTAGtcaaactggaaaaatggcgttg	
	hpa_R	AAGGGGTTATGCTAGtcaaactggaaaaatggcgttg	
intergenic region upstream *asdC*	IR_F	ATCGTGAGGATGCGTactgaacgtgacgctatcag	1300
	IR_R	GCCCTTGCTCACCATttttgactcttttattattagtagg	
* 15bp 5'-overhangs enabling fusion with a plasmid backbone are capitalised.			

**Supplementary Table 2: Compost soil characteristics**	
Classification	Sandy loam with smooth texture
Dry matter content	83.8%
Organic matter content	23,6%
pH	8
2-4 mm particles	1,4%
Larger than 4 mm particles	1,6%

References

1. Wick RR, Judd LM, Gorrie CL, Holt KE. 2017. Completing bacterial genome assemblies with multiplex MinION sequencing. Microb Genom 3. https://doi.org/10.1099/mgen.0.000132

2. Kolmogorov M, Bickhart DM, Behsaz B, Gurevich A, Rayko M, Shin SB, Kuhn K, Yuan J, Polevikov E, Smith TPL, Pevzner PA. 2020. metaFlye: scalable long-read metagenome assembly using repeat graphs. Nat Methods 17:1103–1110. https://doi.org/10.1038/s41592-020-00971-x

3. Antipov D, Korobeynikov A, McLean JS, Pevzner PA. 2016. HybridSPAdes: An algorithm for hybrid assembly of short and long reads. Bioinformatics 32:1009–1015. https://doi.org/10.1093/bioinformatics/btv688

4. Pan S, Zhao XM, Coelho LP. 2023. SemiBin2: self-supervised contrastive learning leads to better MAGs for short- and long-read sequencing. Bioinformatics 39:I21–I29. https://doi.org/10.1093/bioinformatics/btad209

5. Chklovski A, Parks DH, Woodcroft BJ, Tyson GW. 2023. CheckM2: a rapid, scalable and accurate tool for assessing microbial genome quality using machine learning. Nat Methods 20:1203–1212. https://doi.org/10.1038/s41592-023-01940-w

6. Aroney STN, Newell RJP, Nissen JN, Camargo AP, Tyson GW, Woodcroft BJ. 2025. CoverM: read alignment statistics for metagenomics. Bioinformatics 41. https://doi.org/10.1093/bioinformatics/btaf147

7. Li W, O'Neill KR, Haft DH, Dicuccio M, Chetvernin V, Badretdin A, Coulouris G, Chitsaz F, Derbyshire MK, Durkin AS, Gonzales NR, Gwadz M, Lanczycki CJ, Song JS, Thanki N, Wang J, Yamashita RA, Yang M, Zheng C, Marchler-Bauer A, Thibaud-Nissen F. 2021. RefSeq: Expanding the Prokaryotic Genome Annotation Pipeline reach with protein family model curation. Nucleic Acids Res 49:D1020–D1028. https://doi.org/10.1093/nar/gkaa1105

8. Chaumeil PA, Mussig AJ, Hugenholtz P, Parks DH. 2020. GTDB-Tk: A toolkit to classify genomes with the genome taxonomy database. Bioinformatics 36:1925–1927. https://doi.org/10.1093/bioinformatics/btz848

9. Choi K-H, Schweizer HP. 2006. mini-Tn7 insertion in bacteria with single attTn7 sites: example *Pseudomonas aeruginosa*. Nat Protoc 1:153–161. https://doi.org/ 10.1038/nprot.2006.24
